# Stable acetate production in extreme-thermophilic (70°C) mixed culture fermentation by selective enrichment of hydrogenotrophic methanogens

**DOI:** 10.1038/srep05268

**Published:** 2014-06-12

**Authors:** Fang Zhang, Yan Zhang, Jing Ding, Kun Dai, Mark C. M. van Loosdrecht, Raymond J. Zeng

**Affiliations:** 1 Department of Chemistry, University of Science and Technology of China, Hefei, Anhui 230026, People's Republic of China; 2 Hebei Key Laboratory of Applied Chemistry, School of Environmental and Chemical Engineering, Yanshan University, Qinhuangdao, Hebei 066004, People's Republic of China; 3 Department of Biotechnology, Delft University of Technology, Julianalaan 67, 2628 BC Delft, The Netherlands

## Abstract

The control of metabolite production is difficult in mixed culture fermentation. This is particularly related to hydrogen inhibition. In this work, hydrogenotrophic methanogens were selectively enriched to reduce the hydrogen partial pressure and to realize efficient acetate production in extreme-thermophilic (70°C) mixed culture fermentation. The continuous stirred tank reactor (CSTR) was stable operated during 100 days, in which acetate accounted for more than 90% of metabolites in liquid solutions. The yields of acetate, methane and biomass in CSTR were 1.5 ± 0.06, 1.0 ± 0.13 and 0.4 ± 0.05 mol/mol glucose, respectively, close to the theoretical expected values. The CSTR effluent was stable and no further conversion occurred when incubated for 14 days in a batch reactor. In fed-batch experiments, acetate could be produced up to 34.4 g/L, significantly higher than observed in common hydrogen producing fermentations. Acetate also accounted for more than 90% of soluble products formed in these fed-batch fermentations. The microbial community analysis revealed hydrogenotrophic methanogens (mainly *Methanothermobacter thermautotrophicus* and *Methanobacterium thermoaggregans*) as 98% of Archaea, confirming that high temperature will select hydrogenotrophic methanogens over aceticlastic methanogens effectively. This work demonstrated a potential application to effectively produce acetate as a value chemical and methane as an energy gas together via mixed culture fermentation.

As a mature and well-studied technology, mixed culture fermentation (MCF) is ubiquitously implemented for converting municipal, industrial and agricultural organic wastes into biogas, which offers the benefits of environmental protection and energy recovery^1^,^2^. Recently, there has been much attention to the production of the intermediate hydrogen gas (Eq.1 and Eq.2) as it is a renewable, high weight based energy density and carbon-free energy source^3^,^4^,^5^. The production of chemicals and/or bioenergy from waste is however a more sustainable option for waste handling^2^,^6^. The total conversion of organic wastes to acetate and hydrogen (Eq.1) is an attractive option. However, the low hydrogen yield (commonly lower than 2 moles hydrogen per mole glucose) limits its application^1^. Besides H_2_ yield, the production of volatile fatty acids (VFAs) also has several restrictions. VFAs, a mixture (as shown in Eq.1 and Eq.2) including acetate and butyrate, etc., have to be separated and purified before their utilization. Therefore, the production of a single VFA at elevated concentration in liquid is highly desirable for downstream processing. 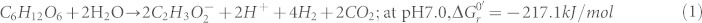


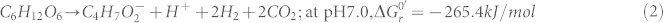


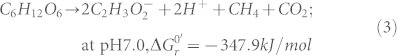
The hydrogen partial pressure (P_H2_) is one of the critical factors for the fermentation product spectrum^7^,^8^. Thermodynamically, acetate as the sole metabolite in liquid (Eq. 1) can only occur at P_H2_ below 60 Pa^8^. Compared to the energy-intensive operations such as gas striping and gas separation by membrane, methanogenesis from hydrogen and CO_2_ by hydrogenotrophic microorganisms is a more efficient pathway to reduce P_H2_ below 60 Pa^5^,^9^. Homoacetogenesis is also a possible pathway to consume hydrogen and generate acetate in anaerobic digestion, but the H_2_ threshold for acetate production is 250 Pa under mesophilic (35°C) conditions, which is high compared to the thresholds of 60 Pa^10^. Hydrogenotrophic methanogens can be used to reduce the hydrogen partial pressure to realize stable and efficient acetate production in mixed culture. In this way acetate can be produced as a value chemical from liquid phase and methane as an energy gas from gas phase. Acetate is an important raw material in industry for example in the production of vinyl acetate (PVA) monomer and acetic anhydride^11^.

The production of sole acetate in liquid solution together with methane was firstly proposed by Pavlostathis et al.^12^ using pure co-cultures of *Ruminococcus albus* (Bacterium) and *Methanobrevibacter smithii* (Archaea). Compared to co-culture fermentation, mixed culture fermentation has the advantage of absence of sterilization requirements, stable operation when designed on proper ecological selection principles, potential for stable continuous operation and an adaptive capacity to variations in feed or conditions^6^. This fermentation has not been reported in mixed culture fermentation. The main reason is the difficulty to separate hydrogenotrophic methanogens from aceticlastic methanogens in MCF. Recently, several researches implicate that the temperature can be a potential factor for the enrichment of hydrogenotrophic methanogens over aceticlastic methanogens^13^,^14^. For example, Krakat et al.^14^ reported that hydrogenotrophic methanogens contributed more to methane production than aceticlastic methanogens in MCF at 60°C, while Thauer et al.^13^ found that many hyperthermophilic species are hydrogenotrophic methanogens. A few studies have focused on extreme-thermophilic mixed culture fermentation (at 70°C) for the benefits of the high substrate degradation rate, efficient heat utilization of some wastewater, and better pathogens destruction, etc.^15^. In a biorefinery concept, high temperature also could enhance ammonium removal from the reactor liquid by gas stripping with biogas and then ammonium could be recovered and valorized.

This study aimed to investigate efficient acetate production in extreme-thermophilic (70°C) mixed culture fermentation. The work used: (i) a long-term operation of a continuous stirred tank reactor (CSTR); (ii) determining the yields of metabolites and the acetate purity in bulk solution of CSTR; (iii) evaluating the metabolite shift and the maximum acetate yield in batch and fed-batch reactors; and (iv) analyzing the microbial community of Bacteria and Archaea in CSTR. It is expected that this study may contribute to the development of future waste based biorefineries.

## Results

### The high fraction acetate production in extreme-thermophilic CSTR

**Figure 1:**
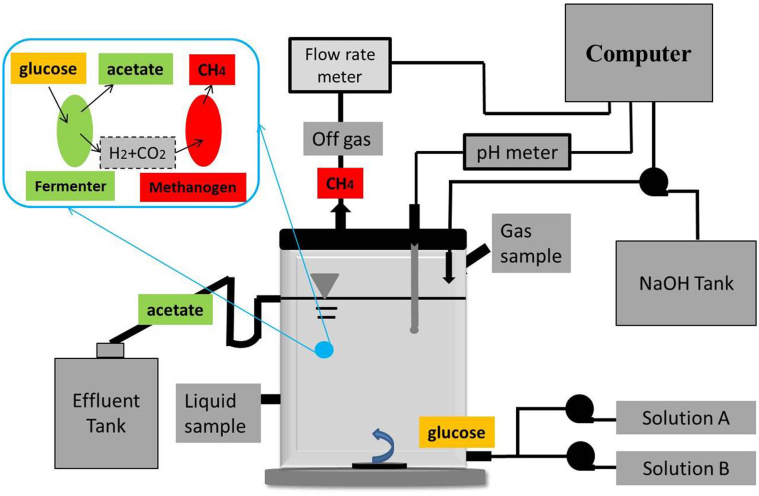
The setup of CSTR for the production of high fraction acetate.

**Figure 2:**
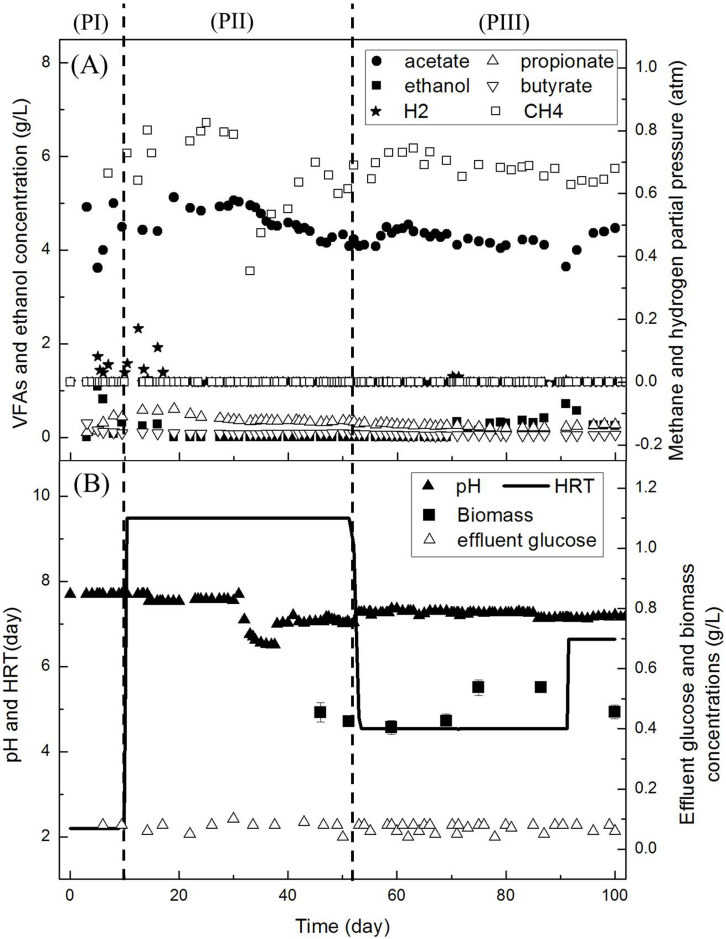
The performances of CSTR for high fraction acetate production.

To investigate production of stable acetate in extreme-thermophilic MCF and determine the yields of metabolites and the acetate purity in bulk solution, a CSTR (as shown in Figure 1) was operated for 100 days under stable conditions. The time course for the operation of the extreme-thermophilic mixed culture fermentation for acetate together with methane production is shown in Figure 2. Obviously, the system was stable, acetate and methane dominated the metabolites during the 100-day operational period. The effluent glucose concentration was always below 0.1 g/L. Meanwhile, lactate, formate and butanol in liquid solution were not detected during the whole operational period.

In period I (PI, day 1–10), the reactor was not stable for acetate production when the HRT (and SRT) was about 2.2 days and pH was 7.5. As shown in Figure 2A hydrogen partial pressure (P_H2_) was about 0.1 atm at day 5, ethanol and propionate also increased to 1.1 and 0.31 g/L, respectively, while acetate decreased from 4.9 to 3.6 g/L. Methane partial pressure (P_CH4_) was about 0.72 atm at day 10, and its production rate was equal to 0.76 L/(L**·**day). The butyrate concentration reduced to 0.09 g/L at day 10.

To ensure the consumption of hydrogen by hydrogenotrophic methanogens and reduce the ethanol and propionate accumulation in liquid solution, HRT in period II (PII, day 11–52) was gradually increased to about 9 days (Figure 2B). The hydrogen content in the headspace and ethanol concentration in liquid gradually decreased to 0 at day 19. Meanwhile, the acetate concentration also increased to 5.1 g/L and the propionate concentration increased slightly to 0.60 g/L. After that, the concentrations of acetate and propionate remained around 5.0 and 0.36 g/L, respectively.

It has been suggested that an elevated bicarbonate concentration at neutral pH may favor propionate production^16^. To reduce propionate accumulation, at day 30, pH was decreased from 7.5 to 6.5 (Figure 2B). However, the propionate concentration remained at about 0.36 g/L, and the acetate concentration decreased from 5.0 at day 30 to 4.5 g/L at day 38. Therefore, pH was increased back to 7.0 at day 38 and controlled between 7.0 and 7.1 for remainder of the experimental period (Figure 2B). P_CH4_ decreased from 0.79 atm to 0.35 atm as pH dropped from 7.5 to 6.5. In later stage it gradually increased to 0.7 atm with increasing pH. The methane production rate was equal to 0.17 L/(L**·**day) at day 45. Hydrogen and ethanol could not be detected at day 19–52. The butyrate concentration in PII was always below 0.08 g/L. The biomass concentration in the reactor was 0.45 ± 0.03 (n = 3) and 0.42 ± 0.01 (n = 3) g/L at day 45 and 52, respectively. HRT in PII was kept around 8.9 days.

To increase the volumetric production rate of acetate in the CSTR, HRT was gradually reduced from 8.9 to 5.0 days in period III (PIII, day 53–90). The main metabolites did not change much in PIII, the acetate concentration was between 4.0 and 4.3 g/L and P_CH4_ was between 0.65 and 0.75 atm (Figure 2A). The propionate concentrations gradually reduced from 0.30 g/L at day 53 to 0.24 g/L at day 100. The butyrate concentration was always below 0.05 g/L. When the temperature dropped temporary due to a heating failure, ethanol increased to 0.33 g/L at day 71, and the maximal concentration was 0.71 g/L at day 91 (Figure 2A). Therefore, HRT of CSTR was gradually increased to 6.5 day at day 92, and the ethanol concentration reduced to 0.18 g/L at day 100. The biomass concentration remained between 0.41 and 0.53 g/L (Figure 2B). In general, the acetate content was above 90% of the metabolites in the liquid phase. These results were comparable to the co-culture experiments^12^, in which the acetate and ethanol concentrations were 3.1 and 0.35 g/L, respectively.

The COD balances of acetate and methane produced from glucose at day 45, 52, 70, 89 and 100 are summarized in Table 1. These balances closed at 92 to 101%. The good COD balances indicated that main metabolites of glucose were detected and other undetected products were minor. The yields of acetate and methane were 1.5 ± 0.06, and 1.0 ± 0.13 mol/mol-glucose, respectively, which are close to the values according to Eq.3. The deviation for acetate is related to biomass formation. The biomass yield was 0.4 ± 0.05 mol/mol-glucose. The yields of propionate and butyrate were very low at 0.08 and 0.01 mol/mol-glucose, respectively. Ethanol was only accumulated after day 71, and the yield was reduced from 0.17 to 0.03 mol/mol-glucose at day 100. The metabolic yields in this study were comparable to those of Pavlostathis, Miller and Wolin^12^, in which the yields of acetate, methane and ethanol were 1.5, 0.8 and 0.2 mol/mol-glucose, respectively. But, the biomass yield of Pavlostathis, Miller and Wolin^12^ was 0.11 mol/mol-glucose, which was lower than that of our study. These results demonstrated that the temperature at 70°C could realize high fraction and stable acetate production in mixed culture and the microorganisms also had good activity.

### Performance of CSTR effluent in batch reactors

**Figure 3:**
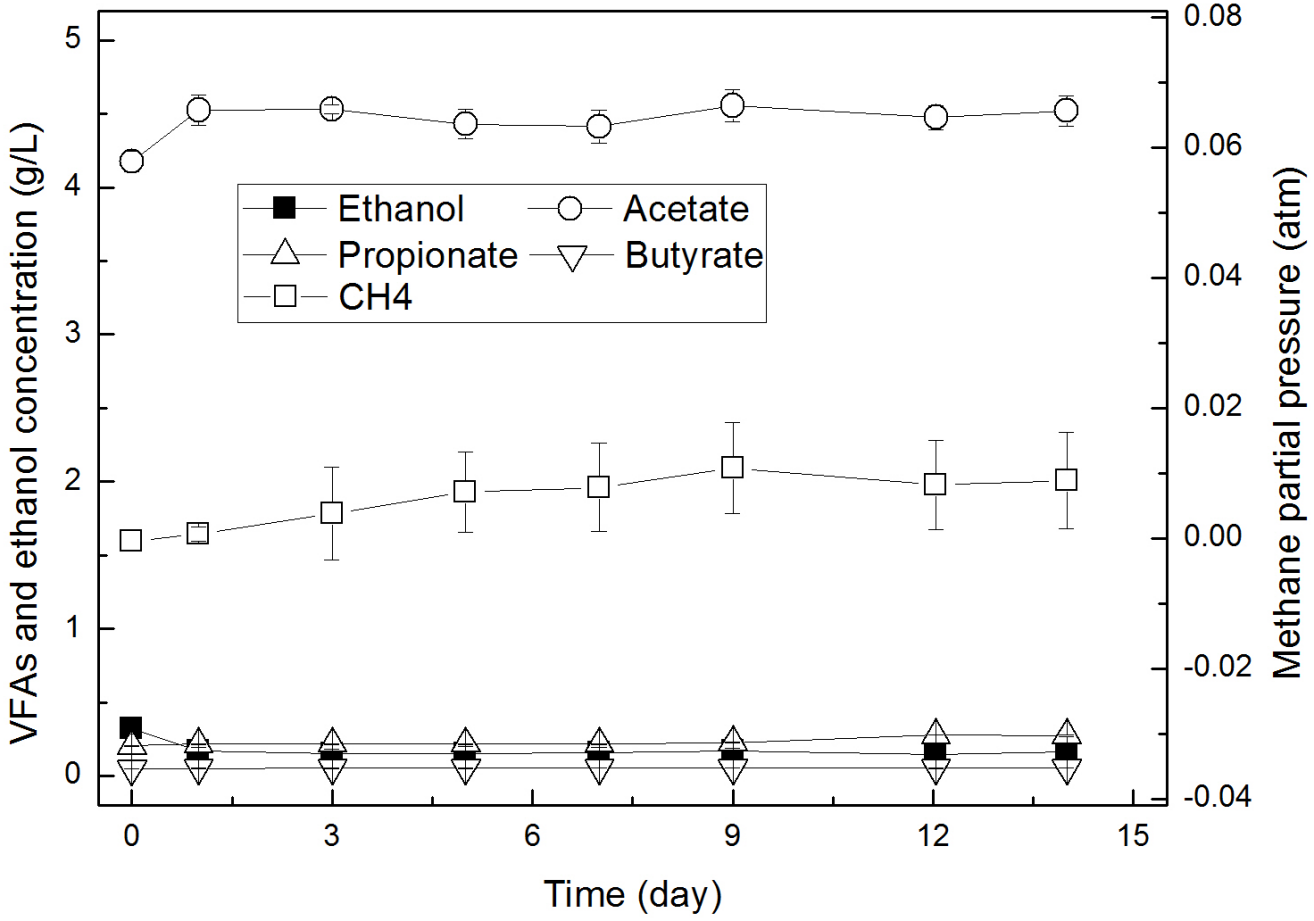
The profiles of VFAs and ethanol from the effluent of CSTR in batch reactors.

To evaluate the metabolite shift in extreme-thermophilic MCF, the effluents of CSTR were collected and the changes of VFAs and ethanol in a batch reactor are shown in Figure 3. Within 14 days, acetate, ethanol, propionate and butyrate all did not change much, which remained at concentrations of 4.4, 0.15, 0.25 and 0.05 g/L, respectively. P_CH4_ did not increase notably either, which remained between 0.007 and 0.01 atm. Other metabolites, including hydrogen, lactate, formate and butanol, were not detected. pH did not change in this work and was 7.5. Therefore, these results strongly indicated that aceticlastic methanogens were not functioning and hydrogenotrophic methanogens were dominating Archaea in the system.

### Production potential for methane and acetate in a fed-batch reactor

**Figure 4:**
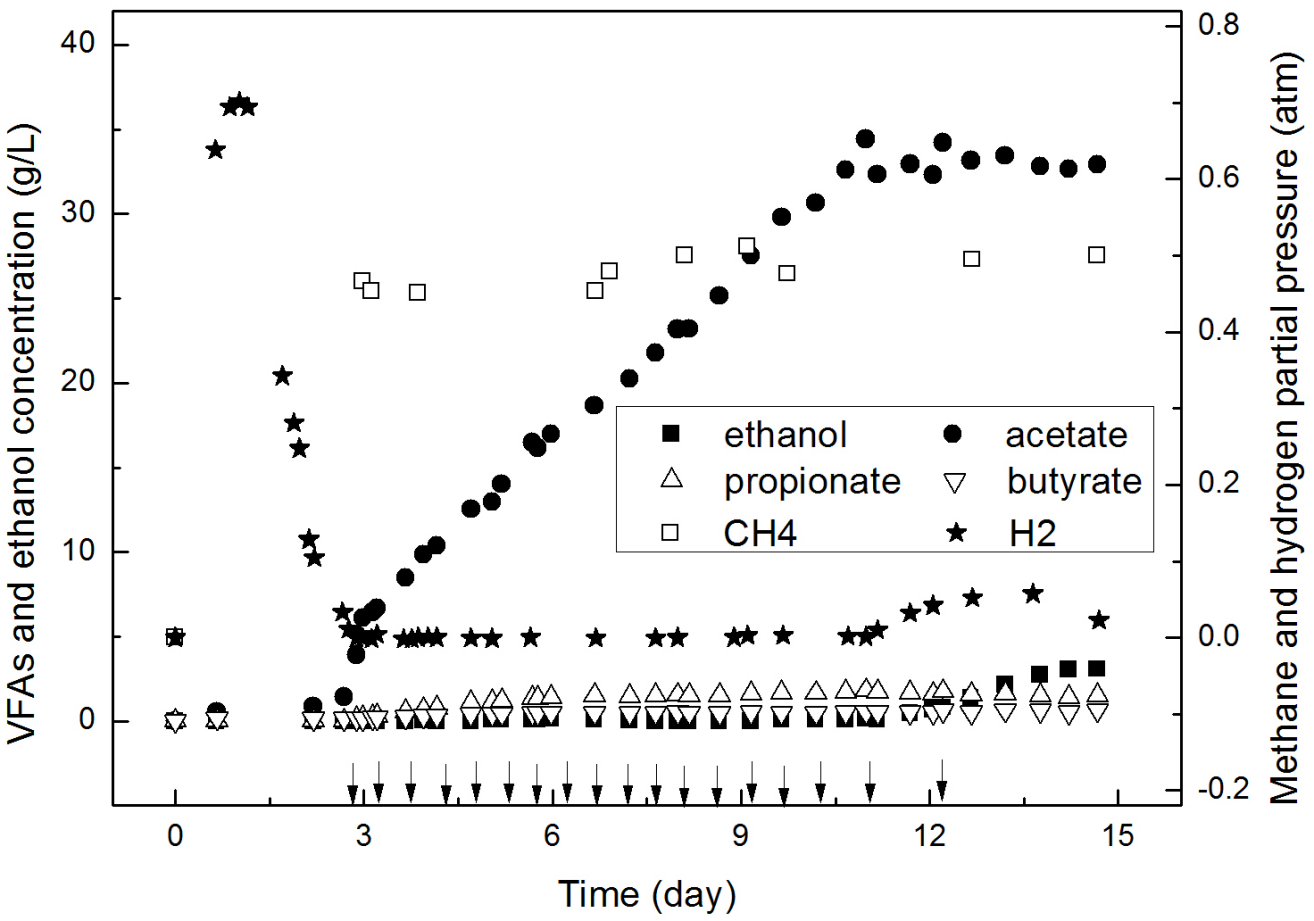
The concentrations of acetate and other metabolites in a fed-batch reactor. Note: ↓ means the addition of glucose.

To evaluate the acetate potential and its yields in extreme-thermophilic MCF, the evolution of metabolites in a fed-batch reactor is shown in Figure 4, in which, acetate also dominated the metabolites of glucose fermentation. pH was maintained at 7.5 during this work. After glucose was added at day 0, acetate and P_H2_ increased to 0.58 g/L and 0.65 atm, respectively. Then P_H2_ reduced notably from 0.7 to 0.0002 atm at day 2.9. Meanwhile, P_CH4_ increased to 0.47 atm. Generally, P_CH4_ was between 0.45 and 0.5 atm at day 4–11. The methane production rate was 1.07 L/(L**·**day), and the yield was equal to 1.1 mol/mol-glucose. After the addition of glucose at day 2.5, acetate increased dramatically from 0.58 g/L to 34.4 g/L at day 11. The acetate production rate was about 3.4 g/(L**·**day) at day 4–11, and the yield was about 1.3 mol/mol-glucose. The concentrations of propionate, ethanol, and butyrate were between 0.11 and 1.5 g/L.

A further glucose addition at day 11 could not increase the acetate concentration anymore (Figure 4). Contrarily, the ethanol concentration increased to 3.1 g/L at day 14.5. Hydrogen accumulated again at day 11.5 and its maximum content was 0.06 atm. The metabolic shift may be due to the acetate inhibition. For example, van Niel, et al.^17^ observed that when the acetate concentration was over 21.9 g/L for *Caldicellulosiruptor saccharolyticus*, it started to inhibit substrate degradation and shift the metabolic distribution. The acetate inhibition resulted in more than 4.0 g/L glucose in the reactor at the end of experiments. The maximum concentration of acetate in our study before day 11 was 34.4 g/L, accounting for 93.8% of liquid metabolites. The acetate concentration and purity were all much higher than those reported for hydrogen producing fermentations^5^. For example, the acetate concentration was just 2.5 g/L and the purity was below 30% when the influent glucose concentration was 18 g/L in CSTR for hydrogen production^5^. Meanwhile, the metabolic yields in the fed-batch reactor were similar to those in CSTR till acetate reached the maximum concentration.

### Microbial community analysis of extreme-thermophilic CSTR

**Figure 5:**
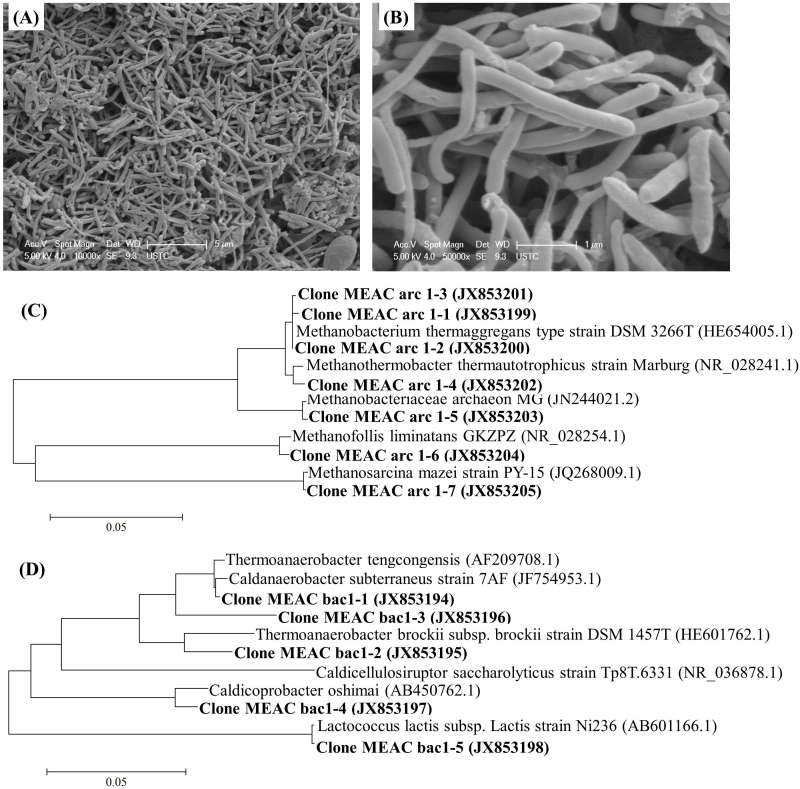
The SEM images of microorganisms in a CSTR, and the Bacteria and Archaea Neighbour-joining trees based on the 16S rRNA gene sequences determined in this study and the related reference sequences. Note: (A) and (B) were the SEM images with scale bar were 5 and 1 μm, respectively. (C) and (D) were the Archaea and Bacteria Neighbour-joining trees, respectively. The scale bar in (C) and (D) indicates 5% sequence divergence. GenBank accession numbers were given in parentheses. The accession numbers of archaea (JX853199-JX853205) and Bacteria (JX853194-JX853298) were the nucleotide sequences in this work.

To analyze the microbial community of Archaea and Bacteria in the CSTR, scanning electron microscopy (SEM) and phylogenetic analysis were conducted. The SEM images in Figure 5A and 5B show that the microorganisms were mainly rod-shape and 3–5 μm long, which were similar to the organisms reported in a thermophilic anaerobic digestion reactor at 60°C^14^. Moreover, as shown in Figure 5C and Table 2, clone MEAC arc1-1, arc1-2, arc1-3 and arc1-4, which were related to the autotrophic microorganisms of *Methanothermobacter thermautotrophicus* and *Methanobacterium thermoaggregans*^18^, dominated the Archaea (their fraction as 91.7%). The percentages of MEAC arc1-5 (related to *Methanobacteriaceae archaeon MG*) and MEAC arc1-6 (related to *Methanofollis liminatans*) that are also autotrophic thermophilic microorganisms^19^,^20^, were rather low at 4.2 and 2.1%, respectively. The percentage of MEAC arc1-7 (related to *Methanosarcina mazei*, the microorganism producing methane from acetate^21^) was just 2.1%. Since autotrophic methanogens accounted for about 98% of Archaea, it was clear that high temperature is enriching for autotrophic methanogens which was crucial for a high fraction acetate production. Meanwhile, these community results matched the reactor performance very well and explained why no acetate consumption occurred within 14 days in the batch experiments.

Figure 5D and Table 3 show the functional Bacteria. Recently, *Thermoanaerobacter tengcongensis*, which was rod-shape, isolated from a freshwater hot spring and can metabolize sugars as principal energy and carbon source, were assigned to the new subspecies of *Caldanaerobacter subterraneus*^22^. As shown in Table 3, *C. subterraneus* and *Thermoanaerobacter tengcongensis* related to clone MEAC bac1-1 and bac1-3, were the dominating bacteria and the percentage was 75.5%. The clone MEAC bac1-4, which was related to *Caldicoprobacter oshimai*^23^, an obligately anaerobic, rod-shape and extremely thermophilic bacterium, was the second dominating bacteria, and the percentage was 20.8%. Other clones including MEAC bac1-2 (related to *Thermoanaerobacter brockii*) and MEAC bac1-5 (related to *Lactococcus lactis subsp.*) were rather low (<1.9%).

## Discussion

### Competition of different metabolic pathways regulated by intermediate H_2_

Fermenting bacteria regulate metabolic distributions in response to the hydrogen partial pressure: at rather low P_H2_, the main metabolites are just acetate, H_2_ and CO_2_; whereas, due to the inhibition of hydrogen at elevated P_H2_, the main metabolites are shifted to ethanol, butyrate, and/or propionate^7^. *Thermoanaerobacter tengcongensis* (namely *C. subterraneus*) was the main fermenting bacterium in this study. Soboh, Linder and Hedderich^8^ found that when P_H2_ in a fermenter increased from 10^−4^ atm to 10^−1^ atm, the main metabolites of *Thermoanaerobacter tengcongensis* were shifted from acetate, H_2_ and CO_2_ to ethanol. In our study, hydrogen was not detected in the stable operational period (Figure 2). Rather low P_H2_ would result in simple metabolite distribution which is in agreement with acetate as dominant (over 90%) liquid metabolite in our study.

The low P_H2_ needed for high acetate productivity is achieved biologically (i.e. hydrogen consumption for methane production by hydrogenotrophic methanogens) rather than mechanically (like gas stripping). It was found that even when high N_2_-sparging rates for H_2_ removal were applied, the metabolites were still a mixture of acetate, butyrate and ethanol in liquid solutions^3^,^24^. As shown in Table 4, except hydrogenotrophic methanogenesis, homoacetogenesis is a possible pathway to consume hydrogen and generate acetate in anaerobic digestion under mesophilic (35°C) and thermophilic (55°C) conditions^25^,^26^. However, we did not find functional bacteria for homoacetogenesis under extreme thermophilic conditions (70°C) in this study. The Gibbs free energy of hydrogenotrophic methanogenesis is higher than that of homoacetogenesis under both 25 and 70°C (Table 4), which means that hydrogenotrophic methanogens get much more energy for growth and maintenance than homoacetogens. In theory, 3 mol acetate/mol-glucose could be produced when fermenting bacteria are co-cultured with homoacetogens only. To achieve it, the crucial criterion is to inhibit hydrogenotrophic methanogenesis, which seems impossible at 70°C. Furthermore, the Gibbs free energy of hydrogenotrophic methanogenesis is also higher than that of aceticlastic methanogenesis, thus, hydrogenotrophic methanogens might get more energy for maintenance and growth, which would benefit for the tolerance to environmental stresses, such as acetate shock^27^. Therefore, in terms of thermodynamic evaluation, hydrogenotrophic methanogens are expected to outcompete aceticlastic methanogens and homoacetogens under extreme thermophilic conditions, which consists with the community analysis in this study. For example, Krakat et al. reported that at 55°C, both hydrogenotrophic methanogens and aceticlastic methanogens were present in anaerobic digestion, but the percentage of clones related to hydrogenotrophic methanogens at 60°C increased to 91%^14^. The percentage of hydrogenotrophic methanogens in our study was a much higher and was above 98%.

It is should be noted that though high temperature could inhibit the activity of aceticlastic methanogens, acetate in the effluent also could be consumed by the syntrophic oxidation pathway. For example, Krakat, et al.^28^ demonstrated that acetate and propionate in a fermenter with a long HRT of 26 days were only 7 mM and 1–2 mM, respectively. Therefore, except high temperature (>60°C), low HRT is also critical for the production of high fraction acetate in mixed culture CSTR. Meanwhile, lower HRT also could reduce operational cost in return.

### Perspectives

To improve the energy recovery and useful chemicals production from organic wastes, several concepts have been proposed. Hydrogen producing reactors, such as two-phase anaerobic processes for hydrogen and methane production, or microbial fuel cells for electricity production from VFAs^1^,^4^,^29^, have been a focus of attention. This work for the first time demonstrated production of predominant acetate together with methane in mixed culture extreme-thermophilic reactors, and the results were comparable to the study of pure cultures and also consistent with the theoretical values (Eq.3). This system was also more stable than hydrogen production from MCF in which the metabolites would change with the operational conditions^3^,^5^.

The development of a sustainable and highly effective method for converting organic wastewaters to valuable chemicals, such as methane and acetate, is strongly desired^6^,^11^. Recently, several researchers demonstrated that the produced biogas could be upgraded via adsorption or biological methods^30^,^31^,^32^. The existing natural gas grid could also be utilized for transportation of the upgraded biogas. Due to the high purity (>90% of carbon compounds) and concentration (34.4 g/L) of acetate in bulk solution, it could also be concentrated by several technologies, such as membrane based liquid−liquid extraction, electrodialysis, etc., and utilized as one useful chemical and/or building block^33^,^34^. Recently, Steinbusch, et al.^35^ proposed that acetate can be transformed to the medium-carbon fatty acids that offer much more advantages than hydrogen, such as high energy density, easy storage and transportation.

Generally speaking, the up-flow anaerobic sludge blanket reactor (UASB) shows several advantages over CSTR for the practical applications, such as higher retained biomass, shorter HRT and higher organic loading, etc^36^,^37^. For example, the biomass concentration was 42.2 g/L and HRT was below 1 day in the study of Dong et al.^36^, while the biomass concentration was just about 0.45 g/L and HRT was 5–10 days in this work. Biomass immobilization in biofilms or granules can reduce acetate inhibition while low HRTs can decrease operational cost and increase methane and acetate production rates, this presented approach is expected to achieve better performance in UASB system which requires further investigation. In terms of energy balance of the overall process and techno-economic issues, extreme-thermophilic (70°C) mixed culture fermentation would benefit from high temperature wastewaters such as biomass hydrolysate. Here we used a pure substrate (glucose), future research will have to show if on complex substrates similar stable conversions can be reached. We trust that by applying the similar selective conditions as here the dominant products will be similar.

In summary, for the first time, production of predominant acetate together with methane in extreme-thermophilic (70°C) mixed culture fermentation was studied. The main outcomes were:

- Acetate accounted for more than 90% of metabolites in liquid solutions. The yields of acetate, methane and biomass in CSTR were 1.5 ± 0.06, 1.0 ± 0.13 and 0.4 ± 0.05 mol/mol-glucose, respectively.
- The formed composition of metabolites proved stable in a long term batch test.
- In a fed batch with biomass enriched in CSTR, the maximal acetate that could be reached was 34.4 g/L with equal yields as in the CSTR.
- The microbial community analysis in CSTR demonstrated that hydrogenotrophic methanogens mainly including *Methanothermobacter thermautotrophicus* and *Methanobacterium thermoaggregans* were the dominant methanogens.

## Methods

### The inoculum, reactor setup, and medium of CSTR

Anaerobic sludge collected from a mesophilic UASB reactor for wastewater treatment in a local brewery was used as inoculum. The inoculum was first sieved to break granular sludge and remove sands, and then sparged with nitrogen (>99.99%) for 20 min before usage. The total volume of the reactor (Figure 1) was 2.0 L, and the working volume was 1.25 L. The reactor was firstly operated in a batch mode during 30 days while the temperature gradually increased from 30 to 70°C. Then, the temperature was maintained at 70 ± 1°C by water bath and the HRT of reactor gradually decreased to 2.2 days within 15 days. pH was adjusted with 2 M NaOH. The influent glucose concentration was 9.0 g/L, and the stirring velocity was 300 rpm.

The reactor influent consisted of 50% Solution A and 50% Solution B, as shown in Figure 1. Solution A contained glucose and 1.0 g/L yeast extract, which was autoclaved at 110°C for 20 min. The composition of Solution B (per litre) was, NH_4_Cl, 1000 mg; KH_2_PO_4_, 400 mg; Na_2_SO_4_, 80 mg; KCl, 100 mg; CaCl_2_, 20 mg; MgCl_2_ 6H_2_O, 140 mg; MnCl_2_·4H_2_O, 1.6 mg; CoCl_2_·2H_2_O, 2.4 mg; FeSO_4_·7H_2_O, 6.4 mg; AlCl_3_, 1.0 mg; NaMO_4_·2H_2_O, 0.2 mg; H_3_BO_3_, 0.4 mg; NiCl_2_·6H_2_O, 1.0 mg; CuCl_2_·2H_2_O, 2.2 mg; ZnSO_4_·2H_2_O, 6.4 mg; EDTA (Na^+^), 6.0 mg.

### Batch and fed-batch experiments for high fraction acetate production

Batch experiments for determining the consumption of acetate and other metabolites with CSTR effluent were performed in three serum bottles (B1, B2 and B3). The total volume of serum bottle was 120 mL and the working volume was 60 mL. After addition of 60 mL effluents, the bottles were sparged with nitrogen (>99.99%) for 20 min and then sealed with butyl-rubber stopper and aluminum cap. The hydrogen and methane contents in the headspace and the concentrations of acetate, ethanol, propionate, butyrate and other metabolites in liquid solution were determined once every two days.

Experiments to determine the maximum yields of acetate and methane were performed in a fed-batch reactor same to the CSTR one. The working volume was also 1.25 L. The fed-batch reactor was inoculated with the concentrated effluents of CSTR to make the initial concentration of biomass was about 2.0 g/L. pH was controlled at 7.0. This reactor was initially sparged with nitrogen (>99.99%) for 20 min. 1.0 g-glucose/L was added at day 0 and 2.0, respectively, and then 6.0 g-glucose/L was added at day 2.5, 2.9, 3.2, 3.7, 4.2, 4.8, 5.2, 5.8, 6.2, 6.8, 7.2, 7.7, 8.2, 8.7, 9.2, 9.8, 10.2, 11.2 and 12.2, respectively. 1.0 g-yeast extract/L was added at day 0, 4.2, 7.2, 8.7 and 11.2, respectively. Metabolite and glucose concentration in liquid solution were determined at least once every two days.

### Analysis of metabolites

The concentrations of VFAs, ethanol and butanol were measured by a Gas Chromatograph (Agilent 7890, CA) with a flame ionization detector and a 10 m × 0.53 mm HP-FFAP fused-silica capillary column. The column operating temperature profiles were 70°C for 3 min, then 10°C/min to 180°C, hold for 4.5 min. The injector and detector temperatures were 250°C and 300°C, respectively. The samples were filtered with 0.45 μm microfilter membrane and then acidified with 3 w/v% formic acid before analysis.

Lactic and formic acids were measured by a high performance liquid chromatograph (HPLC, Agilent 1101, USA) with an ultraviolet (at 210 nm) detector and a C18 reversed phase chromatographic column. The mobile phase contained 97.5 v/v% H_3_PO_4_ solution (1 mL 99.5% HPLC grade H_3_PO_4_ in 1 L deionization water, pH = 3.0) and 2.5 v/v% HPLC grade acetonitrile. Glucose concentration was measured by the phenol-sulfuric acid method^38^.

The produced biogas was recorded daily by a gas meter. The content of hydrogen in the headspace was determined with a Gas Chromatograph (Lunan model SP7890, CN) equipped with a thermal conductivity detector and a 1.5 m stainless steel column packed with 5 Å molecular sieve. The temperatures of the injector, detector, and column were kept at 80, 100, and 50°C, respectively. Nitrogen was used as the carrier gas. The contents of methane and carbon dioxide in the headspace were analyzed with the same gas chromatograph, but the temperatures of the injector, detector, and column were kept at 170, 170, and 150°C, respectively. Hydrogen was used as the carrier gas.

Mixed liquor volatile suspended solid was determined as the biomass concentration by the standard method^39^. The COD balance calculation was based on the COD of each metabolite concentration^5^. Among them, the biomass composition was considered to be CH_1.8_O_0.5_N_0.2_^3^.

### Scanning electron microscopy (SEM) images of biomass

A biomass sample was collected from the reactor effluent and used for SEM analysis (SIRION200, FEI, USA) as follows: the sample was firstly immersed in 5% glutaraldehyde at 4°C for 12 hours, and then was gradually dehydrated in 30%, 50%, 70%, 80%, 95% and 100% ethanol solutions for 15 min each, at last was immersed in 100% 1,1,1,3,3,3-Hexamithyldisilazane solution for at least 30 min.

### DNA isolation and PCR amplification

After CSTR experiment at day 100, biomass was collected at day 101 from the effluent of CSTR and washed with the phosphate buffered saline solution (PBS solution). DNA was extracted from PBS solution. The composition of PBS solution (in 1.0 L distilled water) was NaCl, 8 g, KCl, 0.2 g, Na_2_HPO_4_ 1.44 g, and KH_2_PO_4_, 0.24 g, the pH was 7.4. DNA was extracted from the collected biomass solutions using the DNA Isolation Kit according to the instructions of manufacturer (Biocolor, Shanghai, China). DNA was amplified using the Bacteria 16S rRNA primers: 27F and 1492R, the Archaea 16S rRNA primers: 20F and 958R, respectively^40^. The Amplification was carried out with the following program: 94°C for 2 min, then 35 cycles of 94°C for 30 s, 57°C for 40 s, and 72°C for 2 min, followed by a final extension at 72°C for 10 min.

### Clone library construction and sequencing

Methods of clone library construction and sequencing were according to Thomsen et al.^41^, which were briefly described as: the amplicons were visualized on 1.2% agarose gels, the bands were cut out and then recovered directly with the gel DNA recovery kit (Sangon, Shanghai, China). The recovered products were ligated into a pMD19-T vector (TAKARA, Dalian, China) and then transformed into *Escherichia coli* JM109 competent cells according to the manufacturer's directions. The white clones were obtained by the blue-white screening and were checked by the PCR methods using the M13F and M13R primers. The positive clones were selected and sequenced by the ABI PRISM™ 3730XL DNA Analyzer (Life Technologies Corporation, Shanghai, China).

### Phylogenetic analysis

The above obtained 16S rRNA sequences were compared with sequences in the GenBank database using the NCBI Blast search program (http://blast.ncbi.nlm.nih.gov/Blast.cgi). Closest cultured and uncultured relatives were retrieved from the database. A neighbour-joining tree was made based on the 16S rRNA gene sequences determined in this study and related reference sequences. Alignment and phylogenetic analysis were performed with the MEGA 4.1 (Beta) software. Sequences retrieved in this study were accessible under the accession numbers: JX853199-JX853205 (Archaea) and JX853194-JX853298 (Bacteria).

## Author Contributions

The research was conceived by F.Z. and R.Z.; F.Z. and Y.Z. performed the reactor experiments; F.Z., J.D. and D.K. performed molecular analysis; F.Z., M.C.M.L. and R.Z. contributed to interpret the data and write the paper. All authors reviewed the manuscript.

## Figures and Tables

**Table 1:** The COD balance and metabolic yields of high fraction acetate and methane production from glucose in CSTR experiments

		The yields of metabolites and biomass (mol/mol-glucose)					
Time (day)	COD balance (%)	Acetate	Butyrate	Propionate	Ethanol	Methane	Biomass
45	100	1.52	0.02	0.09	0	1.14	0.38
52	100	1.44	0.02	0.09	0	1.22	0.35
70	93	1.51	0.01	0.07	0	0.99	0.36
89	98	1.49	0.01	0.06	0.17	0.89	0.47
100	100	1.61	0.01	0.07	0.03	0.98	0.40

**Table 2:** Phylogenetic affiliation and clone numbers of Archaea 16S rRNA genes from a CSTR producing methane and acetate

Clone name	Closest relative 16S rRNA phylotypes	Similarity (%)	Clone number	Percent (%)
MEAC arc1-1	*Methanobacterium thermaggregans*	99	5	10.4
MEAC arc1-2	*Methanobacterium thermaggregans*	100	1	2.1
MEAC arc1-3	*Methanobacterium thermaggregans*	99	25	52.1
MEAC arc1-4	*Methanothermobacter thermautotrophicus*	99	13	27.1
Sub-total			44	91.7
MEAC arc1-5	*Methanobacteriaceae archaeon MG*	99	1	2.1
MEAC arc1-6	*Methanofollis liminatans*	99	2	2.1
MEAC arc1-7	*Methanosarcina mazei*	99	1	4.2
	Total	48	100	

**Table 3:** Phylogenetic affiliation and clone numbers of Bacteria 16S rRNA genes from a CSTR producing methane and acetate

Clone name	Closest relative 16S rRNA phylotypes	Similarity (%)	Clone number	Percent (%)
MEAC bac1-1	*Caldanaerobacter subterraneus*	99	37	69.8
MEAC bac1-2	*Thermoanaerobacter brockii*	95	1	1.9
MEAC bac1-3	*Thermoanaerobacter tengcongensis*	95	3	5.7
MEAC bac1-4	*Caldicoprobacter oshimai*	98	11	20.8
MEAC bac1-5	*Lactococcus lactis subsp. lactis*	99	1	1.9
	Total		53	100

**Table 4:** the Gibbs free energy of methanogenesis and homoacetogensis reactions

Reaction	ΔG^0′^ at 25°C (kJ/mol)*	ΔG^0′^ at 70°C (kJ/mol)*
hydrogenotrophic methanogenesis	−135.5	−119.6
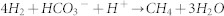		
homoacetogenesis	−104.5	−83.2
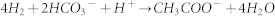		
aceticlastic methanogenesis	−31.0	−36.4
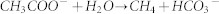		

*: calculated under standard conditions at pH 7.
